# The Effect of Dry Carbon Dioxide Bathing on Peripheral Blood Circulation Measured by Thermal Imaging among Patients with Risk Factors of PAD

**DOI:** 10.3390/ijerph18041490

**Published:** 2021-02-04

**Authors:** Hanna Zbroja, Mateusz Kowalski, Anna Lubkowska

**Affiliations:** Department of Functional Diagnostics and Physical Medicine, Faculty of Health Sciences, Pomeranian Medical University in Szczecin, 71-210 Szczecin, Poland; mateuszk888@gmail.com (M.K.); annalubkowska@gmail.com (A.L.)

**Keywords:** carbon dioxide, cardiovascular disease, peripheral artery disease, therapy, thermography

## Abstract

Peripheral artery disease (PAD) is becoming a serious health problem of present times. It appears crucial to explore therapies that might help to restore blood flow or increase tissue oxygenation. The most effective methods of detecting early-stage changes in blood circulation in the extremities need to be identified. The aim of this study was to identify the effect of carbon dioxide (CO_2_) bathing on peripheral blood circulation measured by thermal imaging among patients with risk factors of PAD and ankle–brachial index (ABI) in the normal range or ABI indicating some or moderate arterial disease (ABI > 0.5). The correlation between surface temperature change and PAD-relevant characteristics was also examined. Forty-six patients who were over 65 years old who had a minimum of two additional PAD risk factors were recruited. A series of ten dry CO_2_ baths was performed. Thermal images were taken before and after the intervention. The CO_2_ therapy caused a significant change in the body surface temperature of many body areas. Numerous moderate correlations between temperature change and health-related characteristics were identified. Therefore, patients with PAD risk factors could benefit from CO_2_ therapy. Improvements in blood flow change the body surface temperature, and these changes could be successfully detected by thermal imaging.

## 1. Introduction

Peripheral artery disease (PAD) is becoming a serious health problem of present times. It is the third most common cardiovascular disease after coronary blood vessel disorders and stroke [1]. Among cardiovascular deaths, heart attack and stroke account for 85% of fatalities. The age-standardized rate (per 100,000) of deaths caused by cardiovascular diseases is 15,616.1, while for PAD, it is 49.8. Peripheral arterial disease is a failure of the proper functioning of arteries outside of the heart and brain. As a result, a reduction of blood circulation occurs in the human body, particularly in the extremities. PAD is most commonly caused by atherosclerosis, where a plaque causes narrowing or obstruction of the arteries. Most PAD patients are asymptomatic, but at the same time, many of them experience intermittent claudication (pain during walking) [2]. Critical limb ischemia occurs when the reduction in blood flow is severe enough to cause pain while resting or to cause tissue atrophy. Peripheral arterial disease is so dangerous that 10–15% of patients with intermittent claudication die within five years of diagnosis [3]. While the cardiovascular disease may not be the main cause of death of these patients, PAD might trigger several health complications that significantly shorten the lifespan of PAD patients. Therefore, it appears crucial to explore therapies that may help to restore blood flow or increase tissue oxygenation.

Carbon dioxide (CO_2_) bathing has been shown to be effective in the treatment of peripheral vascular disorders. The effect of the percutaneous application of CO_2_ has been broadly studied. Carbon dioxide bath effectiveness is based on the fact that carbon dioxide acts as a vasodilator of blood vessels. Vasodilation properties of CO_2_ bathing have been proven by the study examining laser Doppler flux in cutaneous microcirculation in a group of young, healthy participants [4]. After a 35-min CO_2_ therapy, a significant increase in the extremities blood flow has been observed. Intracorporeal variations in CO_2_ concentrations affect both blood flow and the capacity of hemoglobin to release oxygen [5]. Carbon dioxide therapy has been proven to be effective in diminishing hypoxia-related damage, as it causes a significant increase in tissue oxygenation. The other controlled clinical trial with peripheral occlusive arterial disease patients provided proof that CO_2_ bath has a positive effect, reducing free radical plasma levels and increasing antioxidant levels [6]. It has been concluded that CO_2_ baths result in an improvement in microcirculation. Furthermore, a series of everyday CO_2_ baths over four weeks has resulted in elevated arterial peak flow, increased transcutaneous oxygen tension and most importantly longer pain-free walking distance among peripheral occlusive arterial disease patients [7]. This shows that CO_2_ baths can be a clinically effective treatment for PAD patients. The other clinically important result of CO_2_ baths was observed among patients with critical limb ischemia with ulceration or gangrene [8]. After CO_2_ bathing (10 min twice a day for 2 months), 83.1% of limbs became salvageable, and in as many as 96.4% of limbs, there was a reduction of ulcer or gangrene area to only one toe. Taking all the above evidence together, carbon dioxide bathing appears to be a very promising therapy for peripheral arterial disease patients. Therefore, research should focus on identifying the most effective methods of detecting early stage changes in blood circulation in the extremities, so that preventive therapies could be implemented.

Thermal imaging is a non-invasive, diagnostic imaging method [9]. It uses the measurement of medium and long waves of infrared radiation, which are emitted by any object/body whose temperature is above the absolute zero temperature [10]. The recorded wavelength is converted into temperature and presented visually as a thermograph/ thermogram [9]. Specialized software generating a thermal image allows assessment of the surface temperature of both the entire body and region of interest (ROI) [11]. Thermal imaging methods of skin temperature assessment in real time are characterized by measurement accuracy (up to 1%), non-invasiveness, high sensitivity (up to 0.025 °C) and do not require direct physical contact with the patient [12].

Thanks to its properties, thermal imaging measurements are increasingly used in medicine to assess: the effect of cryotherapy, prevention and treatment of sports injuries, brown adipose tissue activity, surface temperature, metabolic disorders and temperature anomalies, inflammation, tumors, the effect of physical activity on temperature or as a method diagnostic of vascular diseases [11,13]. The wide application of thermal imaging in the diagnosis of disorders based on the analysis of the surface temperature of the skin (T_SK_) results from factors influencing its regulation. According to scientific reports, the regulation of skin temperature depends on blood pressure, the amount of subcutaneous tissue, the activity of the autonomic nervous system, and metabolism [12,14]. According to the studies by Lahiri [15] and Ring and Ammer [16], the clinical abnormality and the occurrence of disorders are reflected in an increased or decreased blood flow, which will directly increase or decrease the surface temperature of the skin.

Blood flow through the blood vessels (arteries and veins) is determined by the difference in systolic and diastolic vascular pressure and factors, which will affect the diameter of the blood vessel [17]. These factors include, among others, atherosclerosis [18]. Peripheral arterial disease may be asymptomatic in approximately 40% of patients [19], or it may cause coldness of the lower limbs, exertional pain, which may also appear at a later stage at rest, changes in skin color, and numbness [20]. The ankle–brachial index (ABI) is very often used to diagnose PAD. An ankle–brachial ratio above 0.9 indicates normal blood supply, while ABI < 0.9 indicates clinical signs of circulatory impairment, including ABI < 0.4 is critical ischemia. ABI has been shown to have high specificity (85.7%) and sensitivity (85.3%) for identifying PAD [21,22].

Due to the consequences of PAD and the frequent symptoms of this disease, i.e., coldness of the lower extremities, the use of thermal imaging seems to be a justified management procedure in this disease, as it not only assesses the initial condition of the patient, but also allows to monitor the progress of treatment [20].

In the current literature, the effectiveness of CO_2_ therapy among patients with moderate and severe arterial disease is frequently assessed. However, there is a lack of evidence regarding the effectiveness of CO_2_ therapy among patients with risk factors for PAD, yet their ABI is in the normal range or indicating only some arterial disease. The aim of this study is to identify the effect of carbon dioxide bathing on peripheral blood circulation measured by thermal imaging among patients with risk factors of PAD and ABI in normal range or ABI indicating some or moderate arterial disease. The secondary purpose of this study is to examine correlation between surface temperature change and PAD-relevant characteristics.

## 2. Materials and Methods

The study was approved by the Ethics Committee of the Pomeranian Medical University in Szczecin, Poland (KB-0012/16/17). This was a double-blinded randomized control trial. Recruitment for the study was carried out at the healthcare facilities in Szczecin. The information was promoted by posters and leaflets. The number of participants in each study group was based on the number of available volunteers, who expressed interest in participating in the trial.

Inclusion criteria were as follows:

- Written consent of the patients to participate in the study;
- Over 65 year of age;
- Minimum of additional two PAD risk factors among smoking, diabetes mellitus, hypertension, dyslipidemia, elevated C-reactive protein (CRP), hyperviscosity or hypercoagulable state, hyperhomocysteinemia, chronic renal insufficiency.

Peripheral arterial disease risk factors were defined according to the following criteria:

- Smoking–habit present or not (according to the patient-filled questionnaire);
- Diabetes mellitus–previously diagnosed diabetes mellitus, use of diabetic drug or fasting plasma glucose ≥ 126 mg/dL;
- Hypertension—previously diagnosed hypertension, use of blood pressure-lowering drug or systolic blood pressure ≥ 140 mmHg and/or diastolic blood pressure ≥ 90 mmHg;
- Dyslipidemia—previously diagnosed hypertension, use of a lipid-lowering drug, or any one of the following: total cholesterol level ≥ 240 mg/dL, triglyceride level ≥ 150 mg/dL, low-density-lipoprotein (LDL) cholesterol level ≥ 140 mg/dL, high-density-lipoprotein (HDL) cholesterol level < 40 mg/dL;
- Elevated c-reactive protein—CRP > 3.0 mg/L;
- Hyperviscosity or hypercoagulable state—previously diagnosed hyperviscosity or hypercoagulable state;
- Hyperhomocysteinemia—blood homocysteine concentration > 15 µmol/l; chronic renal insufficiency—previously diagnosed chronic renal insufficiency.

Exclusion criteria were as follows:

- Severe arterial disease defined by ankle–brachial index < 0.5;
- Diagnosis of Buerger’s disease;
- Current chemotherapy or radiotherapy;
- Current therapy with any immunosuppressive agent;
- Planned amputation within 6 months;
- Concurrent participation in another clinical study.

**Figure 1:**
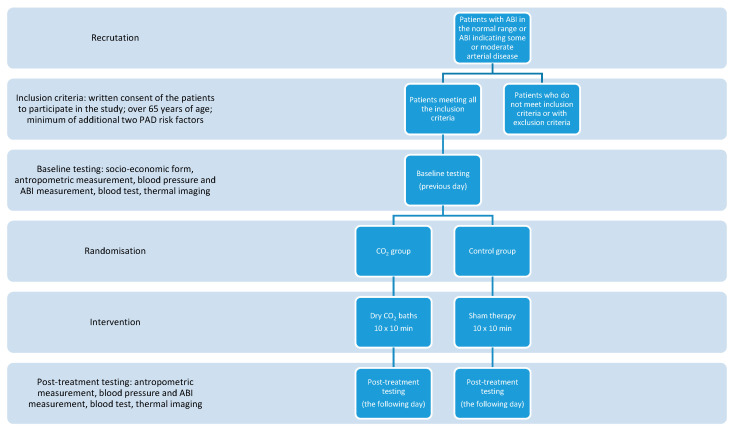
Study design.

All the tests were performed at the Department of Functional Diagnostics and Physical Medicine at the Pomeranian Medical University in Szczecin, Poland. The study design is presented in Figure 1. All the participants were asked to fill in a socioeconomic form. The anthropometric measurements were performed, blood pressure and ABI (using MESI ABPI MD) were measured. The blood tests were also performed in order to identify parameters as follows: fasting glucose, glycated hemoglobin, total cholesterol, HDL cholesterol, LDL cholesterol, triglycerides, C-reactive protein, and homocysteine.

All the participants were randomly assigned to one of two groups: control group (sham therapy) or CO_2_ group (participants were taking a dry CO_2_ bath). The randomization was based on a random number generation using a computer software program. Baths were performed using a MAXimus TOWER MX-1 device (MAXimus, Szczecin, Poland). The device allows for whole-body exposure (excluding the head). During the bath, a participant was sitting in the sealed chamber (neck and head stayed outside of the chamber). In the CO_2_ group, the CO_2_ gas was introduced into the chamber and automatically kept at the required concentration. In the sham, group CO_2_ was not introduced. For a better gas exposure patients were asked to take baths in their underwear. The duration of a single bath was 10 min. A series of 10 treatments was performed over 12 day (five consecutive days, two days off, five consecutive days). The duration of a single bath, as well as the number of treatments was chosen in accordance with the Polish health system guidelines for the dry CO_2_ bathing therapy.

### 2.1. Thermal Imaging Measurements

Thermal images were taken one day before a series of CO_2_ baths and one day after the entire series of treatments has finished. A FLIR T103sc HD camera was used for the tests. This camera uses a detector with a resolution of 024 × 768. The thermal sensitivity of the camera is <0.02 K. All the photos were taken in accordance with the guidelines of the European Thermographic Society. Before taking the picture, the patients acclimatized to the conditions in the room for 10 min. The acclimatization time was measured from the moment the outerwear was removed. During the test, the room temperature was monitored, which was 25 °C, and the air humidity was 50–55%.

In order to obtain reliable, consistent and reproducible results, the TISEM questionnaire was used in the study, the guidelines of which were refined based on studies using thermal imaging [23].

All the participants were subjected to four thermal images in anatomical position in anterior-posterior projections (A-P):

- Frontal plane front upper body;
- Frontal plane front lower body;
- Frontal plane back upper body;
- Frontal plane back lower body.

The following regions of interest were selected for detailed thermal imaging analysis:

- Upper back (UB);
- Lower back (LB);
- Chest (CH);
- Abdomen (AB);
- Upper limb (UL);
- Lower limb (LL).

Additionally, when assessing the distribution of the body surface temperature on the limbs, the following areas were selected for the upper limb (UL):

- arm anterior right (AA_R_);
- Arm anterior left (AA_L_);
- Forearm anterior right (FRA_R_);
- Forearm anterior left (FRA_L_);
- Hand palmar right (HP_R_);
- Hand palmar left (HP_L_);
- Arm posterior right (AP_R_);
- Arm posterior left (AP_L_);
- Forearm posterior right (FRP_R_);
- Forearm posterior left (FRP_L_);
- Hand dorsal right (HD_R_);
- Hand dorsal left (HD_L_).

Lower limb areas:

- Thigh anterior right (TA_R_);
- Thigh anterior left (TA_L_);
- Leg anterior right (LA_R_);
- Leg anterior left (LA_L_);
- Thigh posterior right (TP_R_);
- Thigh posterior left (TP_L_);
- Leg posterior right (LP_R_);
- Leg posterior left (LP_L_).

Each photo taken was saved in a digital form. The FLIR TOOLS program was used for a detailed analysis of the surface temperature distribution. Thanks to it, it was possible to designate symmetrical areas on the body and to compare the obtained results. For each area designated, the program determined the minimum temperature (T_min_), the maximum temperature (T_maks_) and the average temperature (T_mean_). The mean temperature (T_mean_) was used to analyze the results of surface temperature distribution in a given region of interest.

### 2.2. Statistical Analysis

The R-Studio program was used for statistical analysis. The normality of the distribution of the analyzed parameters was determined using the Anderson–Darling test. On its basis, the parametric *t*-test and the nonparametric Wilcoxon test were used for further analyses. A statistical significance level of *p* < 0.05 was adopted for the above statistical analyses.

## 3. Results

The characteristics of 46 participants are presented in Table 1. There was no significant difference between two baseline study groups in any of the measured parameters. The mean age of participants was 71.7 year. Most of the participants were non-smokers or ex-smokers. Among current smokers, the range of smoked cigarettes was from 5 to 40 cigarettes per day. Mean body mass index (BMI) of the group indicates obesity. As many as 26 participants had BMI above 30 kg/m^2^, while 17 subjects were overweight (BMI ≥ 25 kg/m^2^) and only 10 participants had BMI within a normal range. There were no underweight participants (BMI ≤ 18.5 kg/m^2^). Among modifiable PAD risk factors, hypertension and dyslipidemia were the most prevalent. Diabetes mellitus was also considerably prevalent. There was no person with chronic renal insufficiency.

Health-related characteristics of the study group are presented in Table 2. Among health-related characteristics, parameters indicating an increased risk for PAD development were examined. Mean waist-to-hip ratio indicates abdominal obesity among participants. Ankle–brachial index results are also presented in Table 2. Mean ABI was in a normal range for both study groups. One person had ABI above 1.4, which indicates stiffness or calcification of the blood vessels. Eight participants had ABI below 0.9 indicating some arterial disease. The therapy did not trigger improvement in the health-related characteristics. None of the parameters reached the statistically significant change. This is true for both study groups. Post-therapy ABI was also statistically the same as the baseline one.

Furthermore, the effect of dry CO_2_ bathing on body surface temperature was examined (Table 3). Statistical analysis revealed significant change in body surface temperature of leg anterior right and leg posterior right area following the sham therapy. The rest of the regions of interest showed no significant difference in surface body temperature following the sham therapy. The therapy of dry CO_2_ bathing resulted in a significant change of body surface temperature in the majority of the regions of interest, except for arm anterior left, arm anterior right, upper and lower back. In comparison to the control group, body surface temperature following the therapy among the participants of the CO_2_ group was also significantly different for the majority of the regions of interest, except for only the arm anterior right area.

In Table 4 and Table 5, results of the correlation between the change of the surface temperature and health-related characteristics are presented for the control and the CO_2_ group, respectively. These tables present only statistically significant correlations. For the control group, negative correlations were observed between surface temperature change and body fat tissue, homocysteine, waist-to-hip ratio, as well as presence of hypertension and type 2 diabetes mellitus. What is interesting, in two regions of interest of participants in the control group, a positive correlation was identified between surface temperature change and total cholesterol. For the CO_2_ group, statistical analysis revealed a negative correlation between some regions of interest and CRP, fasting glucose and hypercholesterolemia state. On the other hand, a positive correlation was identified for systolic blood pressure and LDL-cholesterol. All the above correlations are moderate.

## 4. Discussion

Cardiovascular diseases (CVD) are the leading cause of death globally [24]. Around 18 million people died from cardiovascular disease in 2016. The social and economic consequences of PAD need to be acknowledged. There is a great need for developing global prevention programs, as well as cost-effective treatments and management policies. Carbon dioxide bathing therapy has been proposed as one of the treatments that may bring health and quality of life-related benefits for patients with hypoxic syndromes and diabetes mellitus. The aim of this study was to identify the effect of carbon dioxide bathing on peripheral blood circulation measured by thermal imaging among patients with risk factors of PAD and ABI in the normal range or ABI indicating some or moderate arterial disease. The correlation between surface temperature change and PAD-relevant characteristics was also examined.

In this study, preventive therapy in the form of dry CO_2_ bathing did not cause a change in health-related characteristics. None of the PAD risk factors were improved. This may be explained by several factors. The size of the study group may be too small to show a significant change. Therefore, a need to perform similar interventions among a greater group of participants would be necessary. The second explanation is that the number of dry baths could be too low to trigger a significant change in health-related characteristics. The reason for choosing only ten dry CO_2_ baths was based on the fact that this is the most prevalent therapy length in the Polish health system. The aim of this study was to examine the effectiveness of the therapy with parameters that are broadly used. The lack of improvement in PAD risk factors indicates a great need for a revision of guidelines for the length of dry CO_2_ bathing therapy.

The main finding of this study is that dry CO_2_ bathing caused a significant change in body surface temperature of lower extremities, as well as in many other body areas. Furthermore, a moderate negative correlation between some regions of interest temperature change caused by dry CO_2_ baths and health-related characteristics, such as CRP, fasting glucose and hypercholesterolemia state, was identified. The negative correlation may be explained by the fact that people with more advanced risk factors achieve smaller benefits from CO_2_ baths. On the other hand, a moderate positive correlation was observed for systolic blood pressure and LDL-cholesterol. Therefore, higher LDL-cholesterol or systolic blood pressure is correlated with a greater change in the leg surface temperature. These results suggest that PAD patients with elevated systolic blood pressure and LDL-cholesterol may be better responders for the CO_2_ bathing therapy. Overall, these results suggest that patients with risk factors of PAD and ABI in the normal range or ABI indicating some or moderate arterial disease can benefit from the CO_2_ bathing therapy. These health improvements result in better circulation that could be successfully observed by thermal imaging.

In the current literature, there are studies supporting our results. The attempt to detect the early stages of foot ulcers among diabetes patients has been done with the thermal imaging method [25]. The temperature of corresponding areas of the right and left feet does not usually differ more than 1 °C in the diabetic foot. In contrast, values for temperature differentials above 2.2 °C indicate possible hyperthermia. In this study, nine out of 85 images have shown hyperthermia areas greater than 1 cm. It has been suggested that this temperature difference may indicate an early stage of ulcer development. However, a prospective study has not been done, and the real clinical significance of these findings is not known.

Similarly, a pilot study among 15 diabetic patients had also the aim of exploring the first signs of diabetic foot disease by high-resolution infrared thermal imaging [26]. However, in this study, the thermal imaging effectiveness in detecting signs of foot ulcers can be assessed, as patients were divided into three groups: patients without present signs of diabetic foot complications, patients with local signs of diabetic foot complications, and patients with diffuse complications. Results revealed that the difference in mean temperature between feet of patients with no complication was at a maximum of 1.5 °C, while for patients with diffuse complication, this difference was at a minimum of 3 °C. Thermal imaging showed that feet with osteomyelitis or Charcot feet are warmer, while feet with critical ischemia were colder compared with the contralateral foot. These results indicate that infrared thermal imaging may be appropriate as a diagnostic tool for the detection of signs of diabetic foot disease. From this study, with the background of previously published literature, temperature difference thresholds for various diabetic foot complications can be drawn [27,28,29]. The temperature difference at the level of 2 °C corresponds to neuropathic ulcers, abundant callus, while 3 °C or more indicates Charcot foot, ulcers with osteomyelitis, as well as critical ischemia.

Most importantly, the thermal imaging method may provide clinically important prevention. A randomized controlled trial lasting for 18 months and involving 225 diabetic patients at high-risk for ulceration was testing the effectiveness of home-based foot skin temperature monitoring in terms of ulcer prevention [27]. Proportional hazard regression analysis revealed that the control group had almost three times higher risk of shorter time to ulceration. Similar results have been achieved in smaller (85 diabetic patients) and shorter studies (6 months) with very similar intervention—home-based foot skin temperature monitoring, but in six (instead of one) predetermined sites of foot [28]. Foot skin temperature monitoring caused a significantly lower risk of diabetic foot complications (5% vs. 20% in the control group). Regular foot skin temperature monitoring results in faster detection of the problem and faster diagnosis of diabetic foot complications. Medical intervention at the time of early stage of developing complications proves to be more effective than treating late-stage diabetic foot complications.

However, studies can also be found that do not support the hypothesis of thermal imaging effectiveness in distinguishing blood flow. For example, a study examining 100 critically ill patients with vascular impairments has shown that patients with or without underlying vascular disease or low perfusion pressures may present with lowered relative sacral temperatures [30]. The purpose of this study was to identify those patients with significant vascular disease and the risk for pressure injury of the sacral area. Thirty-two patients showed temperature differentials that met or exceeded the −1.5 °C threshold between the sacral and the control areas. The average temperature differential was −1.92 °C ± 0.62 °C. However, 6 patients had a temperature differential at the level of +1.5 °C or more. Various clinical subgroups were also assessed separately, and no relationship was found between temperature differentials and perfusion for patients with cardiovascular diseases nor patients with a mean blood pressure of less than 60 mmHg. No significant difference was also found among patients with a deep tissue pressure injury. Therefore, these results do not support the use of temperature differentials to detect patients at particularly high risk for pressure injury owing to local blood flow.

In order to further verify the clinical value of infrared thermal imaging, this method was compared with Doppler compression ultrasonography in terms of deep vein thrombosis [31]. The results revealed the sensitivity at the level of 88.3%, the specificity of 65.0%, and a diagnostic accordance rate of 75.0%. Thermal imaging gave 11.7% of false-positive diagnosis and 35.0% of false-negative diagnosis. It was concluded that infrared thermal imaging could be used for detecting deep vein thrombosis and adjunctive diagnostic screening. The combination of thermal imaging and Doppler compression ultrasonography screening could be a high value for early-stage detection and definitive diagnosis of deep vein thrombosis.

One of the strengths of our study is the fact of automated image analysis. Most of the published literature is based on manual image analysis. The computer-based analysis provides higher accuracy, reliability and replicability. Infrared temperature measurements have some limitations as well. Foot temperature may vary from person to person as a result of age-, sex-, health-related differences, as well as environmental factors. A better-designed study could take into account intraindividual temperature patterns and changes prior to determining disease-related changes.

One of the limitations of this study is the lack of power calculation prior to the recruitment. The results obtained from this study confirmed a low statistical power at the level close to 0.3. In order to assess the number of participants needed to achieve significant change as a result of using dry CO_2_ baths, the sample size was estimated. On the basis of the results reflecting the change in average temperature values as a result of the CO_2_ baths, it was estimated that 100 study participants would be appropriate to have power at the level of 0.8. The other limitation of our study may be the recruitment of patients with ABI within a normal range or ABI indicating some arterial disease.

## 5. Conclusions

On the basis of thermal imaging, this study suggests that patients with risk factors of PAD and ABI in the normal range or ABI indicating some or moderate arterial disease could benefit from dry CO_2_ bathing therapy. Improvements in blood flow change the body surface temperature, and these changes could be detected by thermal imaging. In order to reach a clinically significant change shown by the improvement in PAD risk factors parameters, more than ten dry CO_2_ baths are required, as evidenced by this study.

## Figures and Tables

**Table 1:** Population characteristics.

Population Characteristics	
	*n* = 46
Age, years	71.7 (±4.95)
Male: female sex ratio	30:70
White ethnicity, *n* (%)	46 (100)
Smoking status, *n* (%) Current smoker Ex-smoker Non-smoker	5 (10.9) 18 (39.1) 23 (50.0)
body mass index, kg/m^2^	30.1 (±5.9)
Hypertension, *n* (%)	46 (100.0)
Diabetes mellitus, *n* (%)	34 (73.9)
Dyslipidemia, *n* (%)	43 (93.5)
Hyperviscosity or hypercoagulable state, *n* (%)	5 (10.9)
Elevated inflammation markers, *n* (%)	5 (10.9)
Hyperhomocysteinemia, *n* (%)	6 (13.0)
Chronic renal insufficiency, *n* (%)	0 (0.0)
Unless otherwise stated, data are presented as mean (standard deviation).	

**Table 2:** Health-related characteristics.

Health-Related Characteristics	Control Group			CO_2_ Group		
	T_0_	T_1_	*p*-Value	T_0_	T_1_	*p*-Value
Waist circumference, cm						
Men	106.8 (±11.1)	104.8 (±11.1)	0.95	102.9 (±9.5)	103.1 (±7.8)	0.23
Women	93.1 (±14.0)	92.2 (±14.0)	0.92	97.9 (±10.8)	96.9 (±10.9)	0.89
Waist-to-hip ratio						
Men	0.99 (±0.03)	0.97 (±0.02)	0.98	0.96 (±0.05)	0.97 (±0.03)	0.18
Women	0.88 (±0.05)	0.88 (±0.05)	0.40	0.93 (±0.03)	0.93 (±0.03)	0.53
Ankle–brachial index	1.1 (±0.2)	1.1 (±0.2)	0.91	1.0 (±0.2)	1.0 (±0.2)	0.86
Fasting glucose, (mg/dL)	112.1 (±26.8)	110.8 (±22.0)	0.81	114.4 (±29.3)	118.5 (±34.2)	0.59
Glycated hemoglobin, (%)	5.9 (±0.5)	6.0 (±0.6)	0.99	6.1 (±0.7)	6.1 (±0.7)	0.99
Total cholesterol, (mg/dL)	184.7 (±43.3)	184.0 (±37.1)	0.98	180.5 (±41.0)	174.6 (±39.8)	0.98
LDL-cholesterol, (mg/dL)	112.0 (±40.2)	113.4 (±34.7)	0.67	116.1 (±40.4)	110.9 (±40.8)	0.95
HDL-cholesterol, (mg/dL)	62.0 (±16.1)	60.5 (±16.1)	1.00	52.3 (±13.3)	53.5 (±12.4)	1.00
Triglycerides, (mg/dL)	135.2 (±67.9)	133.0 (±68.4)	0.98	166.7 (±102.4)	135.5 (±66.6)	0.99
C-reactive protein, (mg/L)	2.5 (±2.1)	3.0 (±3.6)	0.79	2.6 (±1.8)	4.1 (±4.4)	0.99
Homocysteine, (mol/L)	13.3 (±2.5)	13.4 (±3.2)	0.86	16.3 (±6.1)	15.7 (±5.5)	0.27
Systolic blood pressure, (mmHg)	140.0 (±22.0)	139.7 (±22.7)	0.28	157.8 (±23.3)	153.5 (±19.4)	0.99
Diastolic blood pressure, (mmHg)	72.2 (±10.6)	72.2 (±10.8)	0.84	79.4 (±11.6)	80.0 (±10.5)	0.79

Unless otherwise stated, data are presented as mean (standard deviation). *p*-value was calculated by two-sample *t*-test and Wilcoxon signed-rank test. T_0_—measurements taken before intervention; T_1_—measurements taken after 10 sham or dry CO_2_ baths. HDL—high-density-lipoprotein cholesterol; LDL—low-density-lipoprotein cholesterol.

**Table 3:** Results of tests of statistical surface temperatures of selected region of interest (ROI) during T_0_ and T_1_ for the study group and the control group.

Control Group							CO_2_ Group						*p*-Value
	**T_0_**		**T_1_**				**T_0_**		**T_1_**				
Region of interest					ΔT	*p*-value T_0_ vs. T_1_					ΔT	*p*-value T_0_ vs. T_1_	*p*-value T_1_ placebo vs. T_1_ CO_2_
	$\bar{X}$ temp. (°C)	±SD	$\bar{X}$ temp. (°C)	±SD			$\bar{X}$ temp. (°C)	±SD	$\bar{X}$ temp. (°C)	±SD			
Arm anterior right	31.73	1.15	32.40	1.10	−0.67	0.682	32.13	1.14	32.01	0.12	0.84	0.075	0.205
Forearm anterior right	31.65	1.20	32.70	1.07	−1.04	0.982	31.76	0.87	31.65	0.11	0.78	0.007	0.001
Hand palmar right	28.71	2.91	31.79	1.51	−3.08	0.288	29.47	3.14	28.54	0.93	2.70	0.001	0.001
Arm posterior right	30.33	1.18	31.57	1.20	−1.24	0.973	30.34	1.41	30.22	0.12	1.27	0.001	0.001
Forearm posterior right	31.73	0.88	32.60	1.06	−0.86	0.296	31.69	1.04	31.28	0.41	1.11	0.004	0.001
Hand dorsal right	28.11	3.11	31.66	1.49	−3.56	0.108	29.40	2.65	28.01	1.39	3.10	0.001	0.001
Arm anterior left	31.85	1.27	32.57	1.17	−0.73	0.766	31.88	0.83	31.87	0.01	0.68	0.076	0.044
Forearm anterior left	31.75	1.21	32.73	1.16	−0.97	0.394	31.79	0.88	31.58	0.21	0.76	0.015	0.001
Hand palmar left	28.69	2.64	31.80	1.29	−3.11	0.233	29.85	2.97	28.70	1.14	3.42	0.001	0.001
Arm posterior left	30.17	1.21	31.43	1.06	−1.25	0.860	30.24	1.34	30.17	0.07	1.27	0.001	0.002
Forearm posterior left	31.65	1.19	32.56	1.30	−0.91	0.242	31.43	0.90	31.10	0.33	0.98	0.030	0.001
Hand dorsal left	28.31	2.63	31.31	1.50	−3.00	0.371	28.97	3.31	28.16	0.80	2.70	0.001	0.001
Thigh anterior right	30.04	1.29	32.08	1.27	−2.04	0.511	30.43	1.23	30.20	0.23	1.12	0.001	0.001
Leg anterior right	31.83	0.65	32.81	0.89	−0.98	0.025	32.00	1.15	31.24	0.76	1.05	0.001	0.001
Thigh posterior right	30.62	1.15	32.53	1.15	−1.91	0.079	30.90	1.17	30.30	0.60	1.09	0.001	0.001
Leg posterior right	31.01	0.78	32.28	0.75	−1.27	0.046	31.10	1.08	30.53	0.57	0.76	0.001	0.001
Thigh anterior left	30.24	1.65	32.13	1.30	−1.88	0.408	30.28	1.20	30.00	0.28	1.09	0.001	0.001
Leg anterior left	32.09	1.01	32.81	0.98	−0.72	0.092	31.62	1.38	31.01	0.62	1.02	0.031	0.001
Thigh posterior left	30.62	1.31	32.40	1.15	−1.78	0.175	31.04	1.19	30.59	0.45	1.01	0.001	0.001
Leg posterior left	31.11	0.67	32.25	0.77	−1.14	0.197	30.98	1.13	30.60	0.38	0.82	0.001	0.001
Chest	32.37	1.09	33.32	0.92	−0.95	0.767	32.47	0.83	32.54	−0.08	0.95	0.006	0.011
Abdomen	31.72	1.40	32.79	1.19	−1.07	0.272	31.60	1.10	31.24	0.36	1.08	0.015	0.001
Upper back	32.39	1.27	33.03	1.05	−0.63	0.775	32.40	1.00	32.32	0.08	0.79	0.103	0.034
Lower back	31.93	1.38	32.59	1.40	−0.66	0.507	31.93	1.18	31.71	0.21	0.97	0.214	0.022

*p*-value was calculated by two-sample t-test and Wilcoxon signed-rank test; T_0_—measurements taken before intervention; T_1_—measurements taken after 10 sham or dry CO_2_ baths; ∆T—difference in the temperature between T_0_ and T_1._

**Table 4:** Statistical significance results in the control group at ΔT.

Variable	ROI	*p*-Value	Cor/Rho/tau	Correlation Type
Total cholesterol	TP_L_	0.037	0.481	Pearson’s
	LP_L_	0.046	0.462	Pearson’s
Body fat	LA_R_	0.022	−0.523	Pearson’s
Homocysteine	AP_R_	0.049	−0.456	Pearson’s
Waist-to-hip ratio	TP_R_	0.049	−0.456	Pearson’s
Hypertension	HP_R_	0.039	−0.408	Kendall
Diabetes	AP_R_	0.023	−0.450	Kendall
	LA_R_	0.021	−0.457	Kendall
	LP_L_	0.037	−0.414	Kendall

ROI—region of interest; Cor—Pearson’s correlation coefficient; Rho—Spearman’s correlation coefficient; tau—Kendall’s correlation coefficient; TP_L_—thigh posterior left; LP_L_—leg posterior left; LA_R_—leg anterior right. AP_R_—arm posterior right; TP_R_—thigh posterior right; HP_R_—hand palmar right.

**Table 5:** Results of statistical significance in the CO_2_ group at ΔT.

Variable	ROI	*p*-Value	Cor/Rho/tau	Correlation Type
Systolic blood pressure	AA_R_	0.031	0.450	Pearson’s
	AB	0.022	0.475	Pearson’s
CRP	TP_L_	0.036	−0.438	Spearman
LDL-cholesterol	LA_R_	0.015	0.500	Pearson’s
Fasting glucose	TP_L_	0.034	−0.446	Spearman
	TA_L_	0.048	−0.416	Spearman
	TP_R_	0.039	−0.433	Spearman
	HD_R_	0.034	−0.442	Spearman
Hypercholesterolemia	AP_R_	0.017	−0.428	Kendall
	AP_L_	0.021	−0.413	Kendall
	LA_R_	0.025	−0.402	Kendall
	LB	0.017	−0.425	Kendall

ROI—region of interest; Cor—Pearson’s correlation coefficient; Rho—Spearman’s correlation coefficient; tau—Kendall’s correlation coefficient; CRP—C-reactive protein; LDL—low-density-lipoprotein cholesterol; AA_R_—arm anterior right; AB—abdomen; TP_L_—thigh posterior left; LA_R_—leg anterior right; TP_L_—thigh anterior left; TA_L_—thigh anterior left; TP_R_—thigh posterior right; HD_R_—hand dorsal right; AP_R_—arm posterior right; AP_L_—arm posterior left; LB—lower back.

## Data Availability

The data presented in this study are available on request from the corresponding author.
